# Structure and Spatial Distribution of the Chironomidae Community in Mesohabitats in a First Order Stream at the Poço D'Anta Municipal Biological Reserve in Brazil

**DOI:** 10.1673/031.011.0136

**Published:** 2011-03-25

**Authors:** Beatriz Figueiraujo Jabour Vescovi Rosa, Vívian Campos de Oliveira, Roberto da Gama Alves

**Affiliations:** Laboratório de Invertebrados Bentônicos, Programa de Pós-graduação em Ciências Biológicas- Comportamento e Biologia Animal, Departamento de Zoologia, Instituto de Ciências Biológicas, Universidade Federal de juiz de Fora. 36036-330 Juiz de Fora, Minas Gerais, Brasil

**Keywords:** aquatic insects, Atlantic forest, lotic systems, substrates

## Abstract

The Chironomidae occupy different habitats along the lotic system with their distribution determined by different factors such as the substrate characteristics and water speed. The input of vegetable material from the riparian forest allows a higher habitat diversity and food to the benthic fauna. The main aim of this paper is to verify the structure and spatial distribution of the Chironomidae fauna in different mesohabitats in a first order stream located at a Biological Reserve in the southeast of Brazil. In the months of July, August, and September 2007, and in January, February, and March 2008, samples were collected with a hand net (250 µm) in the following mesohabitats: litter from riffles, litter from pools, and sediment from pools. The community structure of each mesohabitat was analyzed through the abundance of organisms, taxa richness, Pielou's evenness, Shannon's diversity, and taxa dominance. Similarity among the mesohabitats was obtained by Cluster analysis, and Chironomidae larvae distribution through the Correspondence analysis. Indicator species analysis was used to identify possible taxa preference for a determined mesohabitat. The analyzed mesohabitats showed high species richness and diversity favored by the large environmental heterogeneity. Some taxa were indicators of the type of mesohabitat. The substrate was the main factor that determined taxa distribution in relation to water flow differences (riffle and pool). Stream characteristics such as low water speed and the presence of natural mechanisms of retention may have provided a higher faunistic similarity between the areas with different flows. The results showed that the physical characteristics of each environment presented a close relationship with the structure and spatial distribution of the Chironomidae fauna in lotic systems.

## Introduction

The Chironomidae (Diptera) constitute a highly diversified group of aquatic insects frequently occurring in high density in all kinds of liminic ecosystems (Coffman and Ferrington 1984). The larvae of this family contribute to nutrient cycling in aquatic environments by accelerating decomposition through vegetable material fragmentation from the riparian forest (Sanseverino and Nessimian 2008), and constitute food resource for fish (Rezende and Mazzoni 2003), amphibians (Dutra and Callisto 2005), reptiles (Novelli *et al.* 2008), and invertebrates (Walker 1987). They are, therefore, fundamental components in the aquatic trophic net normally occupying different habitats along the lotic system, with their distribution determined by several factors among them substrate characteristics (Sanseverino and Nessimian 2001).

The substrate can be organic or inorganic (Duan et al. 2008), vary the degree of surface roughness, the size of particles, and the content of the organic matter (Taniguchi and Tokechi 2004). Along a stream, substrates are associated with different water flow speeds, which allow a higher environmental heterogeneity in micro and meso-spatial scales (Beisel et al. 1998). Physical factors, as well as food quality and availability, present a close relationship with the structure and distribution of aquatic insects (Merrit and Cummins 1984). The riparian forest provides substrate and food for the benthic fauna through the input of vegetable parts that fall from trees and accumulate over the stream bed (Suriano and Fonseca-Gessner 2004), contributing to higher habitat diversity. The structure and distribution of the biological communities reflect the aquatic system conditions, which make the study of these communities in preserved environments a fundamental step to the elaboration of management and conservation plans for these areas.

The reminiscent areas of Atlantic forest host a significant share of biological diversity, with high levels of endemism in aquatic and terrestrial habitats (Myers at al. 2000). The location of these areas close to urban centers makes their environments more vulnerable to anthropic pressures and its ecosystem consequent degradation (Resende at al. 2002). The main aim of this paper is to verify the structure and spatial distribution of the Chironomidae fauna in different mesohabitats in a first order stream located at the Poço D'Anta Municipal Biological Reserve in the municipality of Juiz de Fora, state of Minas Gerais, Brazil.

## Materials and Methods

The Poço D'Anta Municipal Biological Reserve is a conservation unity constituted by a reminiscent fragment of the Atlantic forest in secondary succession stage and has an area of 277 ha, located in the municipality of Juiz de Fora, state of Minas Gerais, Brazil (21° 44′ 23. 33″ S to 21° 45′ 51. 78″ S and 43° 18′ 29.28″ W to 43° 19′ 9.70″ W). The areas surrounding the Reserve present a high urban growth that leads to an increase in the pressures against its preservation (Souza 2008), the quality of water bodies, and the diversity of existing habitats.

The studied environment is a first order stream located between the coordinates 21° 44′ 35.8″ S to 21° 44′ 30.7″ S and 43° 18′ 50.6″ W to 43° 18′ 53″ W with altitude around 850 m. It is a permanent spring fed stream, whose margins present dense riparian vegetation, with a predominantly sandy bed with rocks of different sizes and large numbers of fallen leaves and branches.

In the months of July, August, and September 2007 and in January, February, and March 2008, samples were collected in each one of the mesohabitats litter from riffles, litter from pools, and sediment from pools using a hand net (250 µM). All collections were carried out in a stretch of low declivity, previously selected, with length of approximately 300 m. In each month, three patches of each mesohabitat, located at different points along the stretch, were individually sampled over a 10 second-period, totalizing 30 seconds by sample/mesohabitat/month. The samples of litter from riffles, litter from pools, and sediment from pools were obtained and analyzed separately.

Aiming to accomplish a general limnological characterization of the studied stretch, water temperature, pH, electrical conductivity, and the level of dissolved oxygen were registered during the collections using a multisensor (Horiba U10). The results obtained refer to an average of three measurements performed at random points located along the stretch. Stream width, water speed, and depth measurements were taken in different parts of the selected stretch. Water speed was obtained through the fluctuator method (Martinelli and Krusche 2007). The separation of the granulometric fraction of the sediment was done with dry samples at room temperature and passed in sieves with different sizes of mesh (2mm, 0.5 mm, 0.25 mm, and 0.05 mm). In order to analyze the concentration of the organic matter of the sediment, a portion of 2.0 grams of the sample was incinerated at 550° C during 4 hours. The result obtained was the difference between the initial and final weight of the sample and expressed in dry weight percentage (Golterman et al. 1978).

In the laboratory, the samples were fixed in formaldehyde solution 4%, and washed in current water using a sieve with a 0.21 mm mesh. Chironomidae larvae were sorted in a stereoscopic microscope and the specimens preserved in 70° GL alcohol. Larvae were individually mounted on slides in Hoyer's medium and identified to the smallest taxonomic possible level, according to Wiederholm (1983), Epler (1992), and Trivinho-Strixino and Strixino (1995).

For each mesohabitat-month, values related to Shannon's diversity index (Magurran 2004), Pielou's evenness (Magurran 2004), richness of taxa and abundance of organisms were calculated for litter from riffles, litter from pools, and sediment from pools. The dominance index value (DI) was calculated according to Kownacki (1971).

In order to measure the effect of mesohabitats on the variation of the fauna structure, the two-way ANOVA parametric test was conducted for the values of richness and diversity indices. The same test was used for the abundance data after they were logarithmized (log x +1) due to the absence of normality (Shapiro-Wilk, p < 0.05). Because the collections were performed in different seasons (dry and rainy) the test also made it possible to evaluate the effect of the temporal variable. These analyses were made using the statistical program BioEstat 5.0 (free version).

The similarity degree among the mesohabitats was tested through Cluster analysis (UPGMA, Morisita coefficient) based on the numerical abundance of taxa. The program used for this analysis was the Past version 1.49 (free version). The Correspondence analysis (CA) was used as an ordination technique among the samples (mesohabitats-months) and the number of Chironomidade larvae in each sample (Henriques-Oliveira 2003). This analysis was performed using a FITOPAC program version 1.6 (free version). Only the dominant (DI >1) and subdominant taxa (1< DI <10) present in the sampled mesohabitats were included in this analysis. A possible taxa preference for a specific mesohabitat was identified through the indicator species analysis proposed by Dufrêne and Legendre (1997). The statistical significance of each species indicator value was calculated using the Monte Carlo test (1000 permutations). This analysis was made with the PC-Ord program version 4.10.

**Figure 1.**
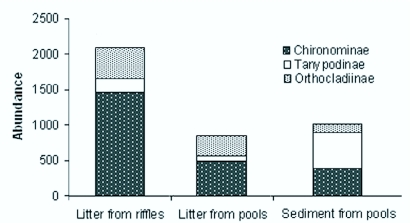
Chironomidae subfamily abundance present in mesohabitats in a first order stream at the Poço D'Anta Municipal Biological Reserve, Juiz de Fora (MG), Brazil. High quality figures are available online.

## Results

**Table 1.**
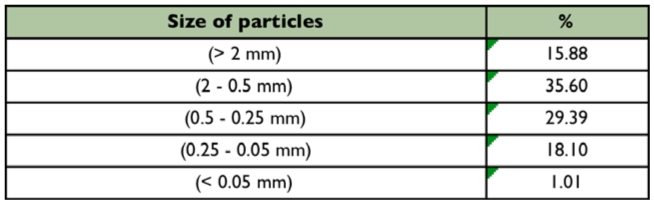
Granulometric characterization of sediment present in a first order stream at the Poço D'Anta Municipal Biological Reserve, Juiz de Fora (MG), Brazil.

The stretch of the studied stream was characterized as narrow, in a shallow sand-bed with predominance of 2 mm to 0.25 mm fractions (Table 1). The waters are transparent, well oxygenated, with low electrical conductivity and slightly acid pH. The average temperature of the water showed little variation during the study. The mean values and respective standard deviation of the environmental variables are presented in Table 2. The average percentage of organic matter of the sediment samples was 30%.

In the analyzed mesohabitats, 3958 Chironomidae larvae were collected, which belonged to three subfamilies, distributed in 35 genera and 12 morphotypes (Table 3). Among the subfamilies, Chironominae were more abundant in litter from riffles (H= 7.20, p <0.05) and litter from pools (H= 4.61, p >0.05), and Tanypodinae were more abundant in sediment from pools (H= 6.53, p <0.05). Orthocladiinae were more abundant in litter from riffles and litter from pools (H= 2.17, p >0.05) (Figure 1). Chironominae presented higher taxa richness in the three mesohabitats analyzed.

**Table 2.**
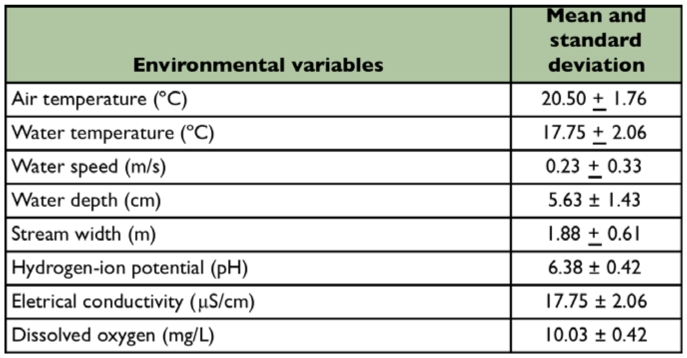
Limnological characterization of the first order stream at the Poço D'Anta Municipal Biological Reserve, Juiz de Fora (MG), Brazil.

**Figure 2.**
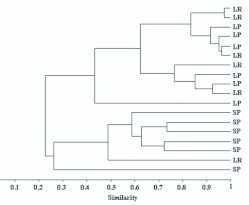
Cluster analysis result of the mesohabitats litter from riffles (LR), litter from pools (LP), and sediment from pools (SP) in a first order stream at the Poço D'Anta Municipal Biological Reserve, Juiz de Fora (MG), Brazil (cofenetic correlation = 0.87). High quality figures are available online.

The dominance index values (DI) indicated that most Chironomidae taxa were adominant in the three mesohabitats (DI <1). In litter from riffles and litter from pools *Rheotanytarsus* and *Paratendipes* (DI >10) were dominant, while *Corynoneura* were dominant only in litter from pools. In sediment from pools, it was indentified dominance of *Larsia*, Pentaneurini and Tanytarsini sp4 (Table 3).

The mean values of Shannon's diversity index, Pielou's evenness, abundance, and total richness (number of taxa corresponding to six months of collection) calculated for each mesohabitat are shown in Table 4.

It was not detected the effect of mesohabitat on taxa richness (F= 0.23; p >0.05; GL = 2), diversity (F = 1.59, p >0.05; GL= 2), nor abundance (F = 2.61; p >0.05; GL = 2). It was not attested the effect of temporal variable (dry and rainy) on richness (F= 3.63; p >0.05; GL = 5), diversity (F= 3.63 p >0.05; GL= 5), nor on abundance (F= 2.69; p >0.05; GL= 5).

**Figure 3.**
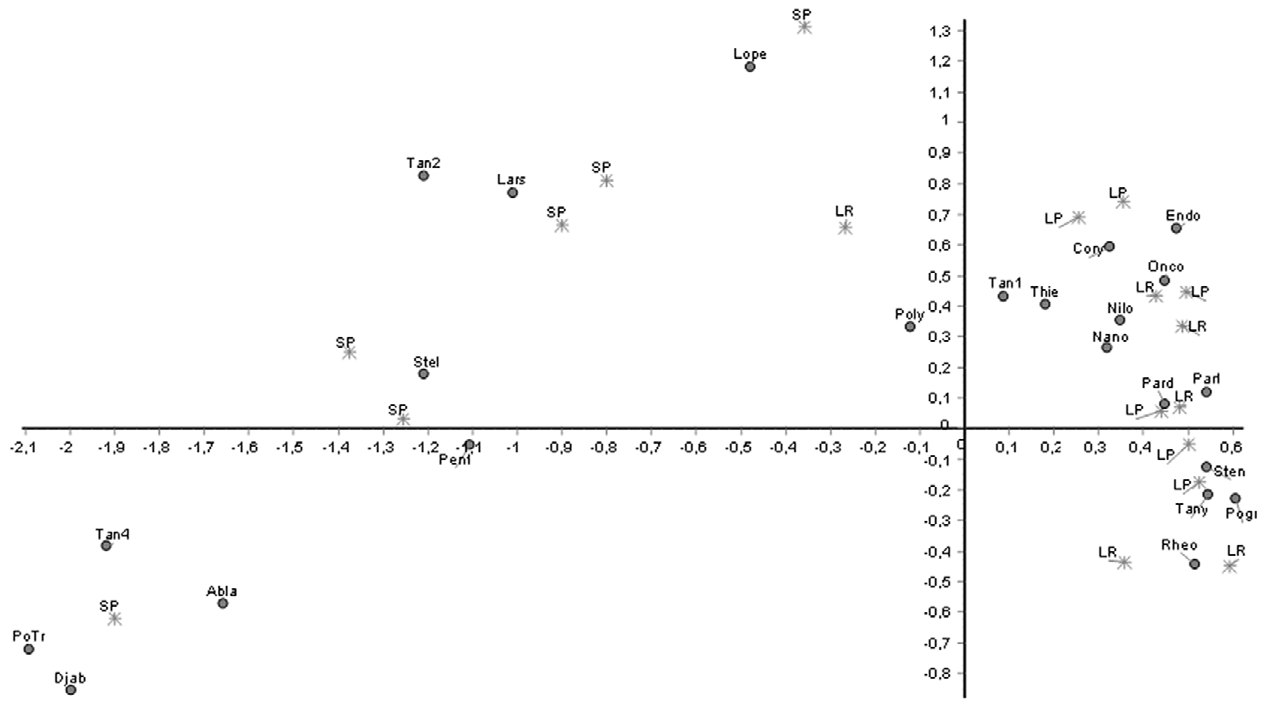
Correspondence analysis (axi I and II) of the samples of each mosohabitat and the distribution of the taxa of Chironomidae in a first order stream at the Poço D'Anta Municipal Biological Reserve, Juiz de Fora (MG), Brazil: Sediment from pools (SP), litter from riffles (LR), and litter from pools (LP). Taxa: (**Abla**-*Ablabesmyia*, **Cory**-*Coryroneura*, **Djab**- *Djalmabatista*, **Endo**-*Endotribelos*, **Lars**-*Larsia*, **Lope**-*Lopescladius*, **Nano**-*Nanocladius*, **Nilo**-*Nilothauma*, **Onco**-*Onconeura*, **Pard**-Paratendipes, **Part**-*Parametriocnemus*, **Pent**- Pentaneurini, **Poly**- *Polypedilum (polypedilum)*, **Potr**-*Polypedilum (Tripodura)*, **Pogr.**- *Polypedilum gr. fallax*, **Rheo**- *Rheotanytarsus*, **Stel**-*Stempellinella*, **Sten**- *Stenochironomus*, **Tan I**- *Tanytasini* sp I, **Tan2**- *Tanytarsini sp2*, **Tan4**-*Tanytarsini* sp4- **Tany**-Tanytarsus, **Thie**- *Thienemannimya*). High quality figures are available online.

Cluster analysis resulted in the formation of two larger groups, according to the nature of the substrate. One group was composed mainly by sediment and the other by litter (Figure 2). The Correspondence analysis result (Figure 3) showed that axis I was responsible for 44.85% of data variation, followed by axis II (14.92%). The analysis indicated that, in the studied stream fauna distribution was more related to the nature of the substrate (litter and sediment) than to differences in water flow (riffle and pool), which reinforces the Cluster analysis result. The indicator species analysis showed that the *Onconeura, Thienemannimya, Endotribelos, Paratendipes*, and *Rheotanytarsus* genera were indicators of litter from riffles, and the *Djalmabatista, Clinotanypus, Harnischia* sp1, *Polypedilum (Tripodura), Stempellinella*, Tanytarsini sp2, and Tanytarsini sp4 were indicators of sediment from pools (Table 5).

## Discussion

The numeric participation of Chironomidae subfamilies varied among the mesohabitats. In litter from riffles, the high abundance of larvae of the Tanytarsini tribe contributed to a higher participation of Chironominae subfamily in this mesohabitat. Tanytarsini larvae are usually abundant in forested streams, especially in current water and litter areas with high presence of particulate organic matter (Sanseverino and Nessimian 2007). In addition, high richness and abundance of Tanytarsisni is to be expected in streams with clean waters and well oxygenated (Helson 2006). In sediment from pool, Tanypodinae larvae were predominant. According to Wiederholm (1983), these larvae are adapted to living in pool areas with soft substrate. The higher abundance of Orthocladiinae larvae observed in litter from riffles and litter from pools in relation to sediment possibly occurred due to the preference of the collected taxa for vegetable substrate.

**Table 3.**
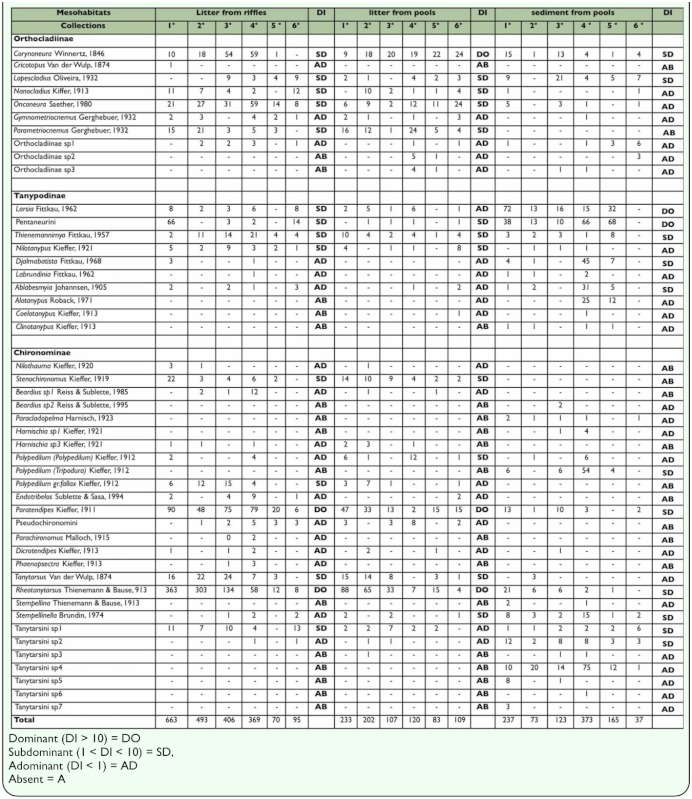
Abundance of Chironomidae and Dominance Index (DI) of the taxa found in litter from riffles, litter from pools and sediment from pool in a first order stream at the Poço D'Anta Municipal Biological Reserve, Juiz de Fora (MG), Brazil.

**Table 4.**
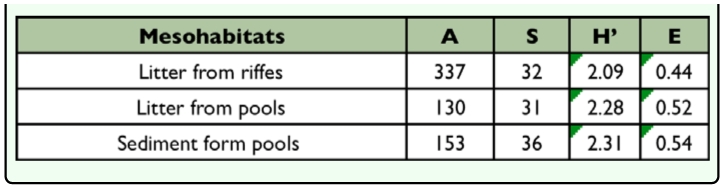
Average abundance (A), richness (S), diversity Shannon's (H') and Pielou's evenness (E) of Chironomidae in mesohabitats in a first order stream at the Poço D'Anta Municipal Biological Reserve, Juiz de Fora (MG), Brazil.

The spatial distribution of aquatic insect larvae is closely related to morphological and physiological adaptations to the physical characteristics of the habitat although some species may exhibit higher plasticity in relation to the type of habitat they occupy (Roque et al. 2007). This is usually observed for various members of Chironomidae family, both in relation to the substrate and to the water flow (Pinder 1995). In this study, the Correspondence analysis showed that some taxa were associated with sediment and others with litter. The *Tanytarsus* genus predominantly occurred in litter, both in riffle and in pool areas, which confirms the results of Sanseverino and Nessimian (2001). However, other authors suggest that these larvae may occupy different types of substrate under different flow conditions (Epler 1992; Rosin and Takeda 2007). The *Polypedilum* genus was present in the three mesohabitats; *Polypedilum (Tripodura)* larvae occurred only in sediment from pools; *Polypedilum gr. fallax* were associated only to litter from riffles; and *Polypedilum (Polypedilum)* larvae were more abundant in litter from pools. Larvae of this genus have a flexible diet, which enables them to occupy different types of substrate under different environmental conditions (Epler 1992; Coofman and Ferrington 1984).

Different from what happens with some taxa of Chironomidae, some others may have higher degree of specialization in relation to the type of substrate they occupy and the water flow (Sanseverino and Nessimian 2001). This fact was observed for the *Onconeura* and *Thienemannimya* genera that were indicators of litter in riffles areas, and for the *Nanocladius, Corynoneura* and *Parametriocnemus* genera which were associated with litter in areas of riffle and pool. This corroborated some studies conducted in tropical streams that found these genera occurring preferentially in litter substrate (Nessimian and Sanseverino 1998; Sanseverino and Nessimian 2001; Henriques-Oliveira et al. 2003; Kikuchi and Uieda 2005). Leaf litter deposited on stream beds is colonized by decomposing microorganisms that increase the appetence of the leaves for invertebrates (Graça et al. 2001). In addition, the heterogeneous structure of this substrate offers better opportunities of shelter for the larvae against the current speed and potential predators (Kikuchi and Uieda 1998). These factors offer suitable conditions for the establishment of the fauna, as it was shown by the high values obtained for richness indices and the high abundance of larvae obtained in the mesohabitats litter from riffle and litter from pool.

The occurrence of taxa in a determined habitat also may be closely related with their feeding habit (Fidelis et al. 2008). The genera *Endotribelos* and *Stenochironomus* which were associated only with litter from riffle mesohabitats, have been found preferably in vegetable substrate, as confirmed in the studies of Roque at al. (2005) and Gallize and Marchese (2007) who attributed this fact to feeding habit shredder and/or mining feeding habit of leaves and wood in decomposition. *Rheotanytarsus*, indicador of the litter from riffles, is a genus usually observed in areas with higher current speed associated with vegetable substrates (Amorim 2004) or to rocks (Henriques-Oliveira 2003). These larvae have tube-building habits which allow their attachment to the substrate in areas with a higher water flow (Hynes 1970) where they take advantage of the water flow to filter food particles (Coffman and Ferrinhton 1984).

**Table 5.**
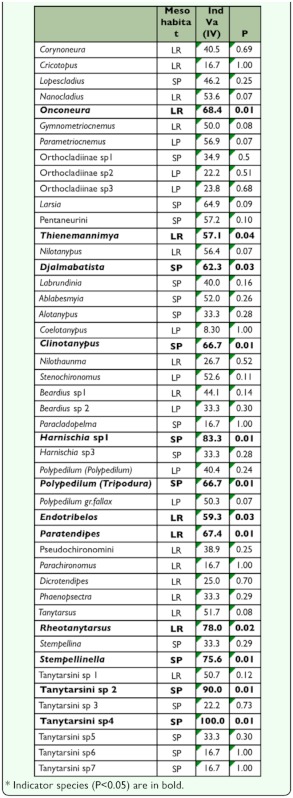
Indicator species analysis result: LR- litter from riffles, LP - litter from pools, SP - sediment from pools.

In contrast to the vegetable substrate, the sandy substrate usually has low diversity and richness in reason of shortage of food resources and refuge for the fauna (Hynes 1970). However, in the studied stream, the high percentage of organic matter present in the sediment in pool areas determined high values of indices for richness and diversity, similar to the vegetable substrate. The vegetable detritus carried by the rains may be retained in pool areas that are formed along the stream by retention mechanisms such as logs fallen on its bed. Therefore, the sediment found in these areas presents a large amount of organic matter which provides favorable conditions to many invertebrate taxa. Furthermore, many Chironomidae more frequently occur in low flow and sandy sediment habitats (Epler 1992), as observed for the taxa *Lopescladius, Larsia, Ablabesmyia*, and Pentaneurini through the Correspondence analysis and for the taxa *Clinotanypus*, Djalmabatista sp2, *Polypedilum (Tripodura)*, Tanytarsini sp2, Tanytarsini sp4, *Harnishia* sp1, and *Stempellinella* through the indicator species analysis.

Most taxa were adominant in the three mesohabitats, a fact that can be attributed to the preserved riparian forest which offers a higher environmental stability and heterogeneity favoring the establishment of a diverse fauna, that is, higher evenness among the groups present in the stream (HenriquesOliveira et al. 1999; Suriano and Fonseca-Gessner 2004). The dominance of *Paratendipes* genera in litter from riffle and pool may be attributed to the good conservation status of the stream, once they are intolerant to damaged environments (Tokeshi 1995) and frequent in forested streams with good quality waters (HenriquesOliveira et al. 1999).

In tropical regions, low-order streams with preserved forest cover receive less influence of different seasons since the marginal vegetation provides a protection, reducing or preventing the impact of rains on the stream bed. This is contrary to what can be observed in higher-order streams or in those streams whose marginal vegetation had been removed, where the effects of the seasons are more accentuated (Henriques—Oliveira 1999; Bispo et al. 2006).

In stretch of the studied stream, it was not observed separation of the fauna in relation to riffle and pool areas. The low average speed of the water (equal to 0.26m/s) (Santos Jr. et al. 2007), the shallow stream depth, and the presence of branches and logs on its bed, possibly contributed to a smaller delimitation of different current speeds and the biota associated with them. Henriques-Oliveira et al. (1999) compared the Chironomidae fauna in two low-order tributaries and observed that the one with dense canopy cover and many natural retention mechanisms, showed smaller amplitude in the flow variation, and higher similarity between the fauna of pools and riffles. In addition, many factors may interact and influence the distribution of the benthic fauna in streams, and the delimitations among the habitats are frequently dynamic (Wallace and Webster 1996), both spatially and temporally that may make it difficult to visualize a pattern for fauna distribution.

Although the differences in water flow speed are among the main variables that determine the distribution of the insect community in lotic systems (Beisel et al. 1998). In the studied stream the nature of the substrate was the main factor that determined the spatial distribution of the fauna. This study reinforces the importance of considering the particular physical characteristics of each environment because they present a close relationship with the structure and the spatial distribution of Chironomidae fauna in lotic systems.
